# Effects of Rationally Designed Physico-Chemical Variants of the Peptide PuroA on Biocidal Activity towards Bacterial and Mammalian Cells

**DOI:** 10.3390/ijms21228624

**Published:** 2020-11-16

**Authors:** Nadin Shagaghi, Andrew H. A. Clayton, Marie-Isabel Aguilar, Tzong-Hsien Lee, Enzo A. Palombo, Mrinal Bhave

**Affiliations:** 1 Department of Chemistry and Biotechnology, Faculty of Science, Engineering and Technology, Swinburne University of Technology, PO Box 218, Hawthorn, VIC 3122, Australia; nshagaghi@swin.edu.au (N.S.); epalombo@swin.edu.au (E.A.P.); 2 Centre for Micro-Photonics, Faculty of Science, Engineering and Technology, Swinburne University of Technology, PO Box 218, Hawthorn, VIC 3122, Australia; aclayton@swin.edu.au; 3 Department of Biochemistry and Molecular Biology, Monash University, Clayton, VIC 3800, Australia; mibel.aguilar@monash.edu (M.-I.A.); john.lee@monash.edu (T.-H.L.)

**Keywords:** antimicrobial peptides, cationic peptides, tryptophan rich peptides, anticancer peptides, puroindoline A

## Abstract

Antimicrobial peptides (AMPs) often exhibit wide-spectrum activities and are considered ideal candidates for effectively controlling persistent and multidrug-resistant wound infections. PuroA, a synthetic peptide based on the tryptophan (Trp)-rich domain of the wheat protein puroindoline A, displays strong antimicrobial activities. In this work, a number of peptides were designed based on PuroA, varying in physico-chemical parameters of length, number of Trp residues, net charge, hydrophobicity or amphipathicity, D-versus L-isomers of amino acids, cyclization or dimerization, and were tested for antimicrobial potency and salt and protease tolerance. Selected peptides were assessed for effects on biofilms of methicillin-resistant *Staphylococcus aureus* (MRSA) and selected mammalian cells. Peptide P1, with the highest amphipathicity, six Trp and a net charge of +7, showed strong antimicrobial activity and salt stability. Peptides W7, W8 and WW (seven to eight residues) were generally more active than PuroA and all diastereomers were protease-resistant. PuroA and certain variants significantly inhibited initial biomass attachment and eradicated preformed biofilms of MRSA. Further, P1 and dimeric PuroA were cytotoxic to HeLa cells. The work has led to peptides with biocidal effects on common human pathogens and/or anticancer potential, also offering great insights into the relationship between physico-chemical parameters and bioactivities, accelerating progress towards rational design of AMPs for therapeutics.

## 1. Introduction

The emergence of multidrug-resistant (MDR) bacterial pathogens and failure of some of the most powerful antibiotics to treat such life-threatening infections have become major concerns worldwide, spurring research on developing novel therapeutics. Antimicrobial peptides (AMPs) are produced as natural defence molecules by many living organisms, from bacteria to humans. AMPs vary widely in their amino acid sequences and secondary structures and typically display broad-spectrum activity against pathogenic bacteria, fungi, viruses and/or protozoa [1]. Further, AMPs can variously act (or be applied) as signalling molecules, immune modulators, mitogens, antitumor agents, contraceptive agents or drug delivery vectors [1,2]. Hence, they are recognised as candidates with great potential to effectively substitute or complement current antibiotics and control MDR microbes. In addition, it is theorised that the emergence of host resistance to AMPs is less likely, as they often act non-specifically on one or more target(s), e.g., the entire bacterial cellular membrane; hence, bacteria may only develop resistance to AMPs with high cost [3]. However, a few reports in the last decade present the development of bacterial resistance to AMPs, by different molecular mechanisms such as changes in cell membrane phospholipids or surface charge, and extrusion of AMPs by active transport efflux pumps [4,5].

Over the past couple of decades, amino acid sequence modifications have been explored to enhance the antimicrobial activity of AMPs and minimize any cytotoxicity to mammalian cells [6,7]. Interdependent molecular determinants such as peptide length, net charge, secondary structure, hydrophobicity, amphipathicity and host membrane composition are all known to significantly affect the degree and spectrum of activity and cell selectivity of AMPs [4,8]; hence, optimisation of such parameters is a powerful tool for designing AMPs. However, many AMPs reported from natural sources have not yet been optimised for large scale use as therapeutics. Additionally, many AMPs are vulnerable to enzyme and salt inactivation, and it is essential to increase their robustness for in vivo and topical applications. However, the correlations of peptide sequences and structures to their biological activity, host cell selectivity, and tolerance to protease digestion or salt in the medium may be peptide-specific and remain poorly researched for a vast majority of reported peptides.

AMPs are commonly classified into subgroups based on a combination of their net charge, secondary structures, origin and amino acid composition. Some AMPs are rich in specific amino acids such as tryptophan (Trp) and arginine (Arg) [6]. The Trp-rich peptides (TRPs), e.g., indolicidin, tritrpticin, lactoferricin and lysozyme, are derived from natural origins and display potent activity against bacteria, fungi, viruses, protozoan pathogens and/or cancer cells [6,9]. This bioactivity has been credited to the unique biochemical properties of Trp that allow the peptide to insert deep into biological membranes [10], enabling or enhancing diverse cell killing mechanisms. This is evidenced through the study of the unique Trp-rich domain (TRD) located in two small, cysteine-rich, antimicrobial proteins called puroindoline A and B (PINA and PINB) found in the wheat seed endosperm [11] which play significant roles in controlling the hardness of the wheat kernels (reviewed in Bhave and Morris 2008; [12]). The TRD is the major determinant of their antimicrobial properties, as confirmed by the antibacterial and antifungal activities of the synthetic peptide PuroA—designed based on it [12,13,14,15]. We have previously designed a number of peptides based on the TRD of PINs encoded by wild type and mutant *puroindoline* gene alleles and related proteins. These synthetic peptides displayed activity against bacteria and phytopathogenic fungi, and certain point mutations affected their activity at quantitative and/or qualitative levels [14]. The Trp-rich peptide PuroA (FPVTWRWWKWWKG-NH2) based on the TRD of the wild-type PINA was found to be active against a number of bacteria and phytopathogenic fungi [14] and fungal spores [15]. Further, studies on other TRPs such as lactoferricin [16] and indolicidin [17] have shown Arg-Trp (RW) motifs to be relevant to the antimicrobial functionality. This was also observed in our earlier work, wherein substitutions of a certain Arg residue, or the Trp residues in PuroA led to major losses of biocidal activity [14]. We have also shown by live cell imaging of cells of the unicellular fungal pathogen, *Candida albicans*, that the mechanism of antimicrobial activity of PuroA involves membrane translocation and binding to intracellular targets, followed by cell perturbation [18]. These observations have led to the present study of optimisation, wherein PuroA has been used as a template to design 13 other peptides with variations in primary structure, length, charge, hydrophobicity, amphipathicity, amino acid isomer content, and dimerisation or cyclisation. Their antimicrobial and anti-biofilm activity, stability in the presence of protease and salt, and toxicity towards mammalian cells, were evaluated, to identify the parameters contributing to the spectrum of their bioactivity.

## 2. Results

The peptide PuroA (FPVTWRWWKWWKG-NH_2_) was designed based on the TRD of wheat seed protein PINA, encoded by the ”wild type” allele Pina-D1a [11] (Genbank accession DQ363911). Its sequence was then used as framework to design 13 other peptides (Table 1). R8 was generated by substituting all non-Trp residues with the cationic Arg. P1 was generated by substituting all uncharged residues with the cationic Arg and Lys, and inserting an additional Trp (Trp4) after Arg3. The shorter peptides R6, R7, W7, W8 and WW, were designed based on the Arg, Lys and Trp of the TRD. A cyclic analog of PuroA with an additional head-to-tail bond in the peptide backbone, and a Lys-linked dimeric C-terminal analog (PuroA)_2_K, were also designed. Three sequences (P1, W7, WW) were further modified to design diastereomeric counterparts (dP1, dW7, dWW) by incorporating 35–40% D-amino acids, the positions of which were selected to allow short consecutive sections of one or two L-amino acids that cannot adopt α-helical structures [19]. The C terminus of all peptides was amidated, except for PuroA-OH.

The theoretically calculated physicochemical properties of the peptides are summarized in Table 1. The designed peptides ranged from 6 to 27 residues in length, a net charge of +2 to +8, and 4 to 10 Trp residues. Three of the peptides were comprised of 35–40% D-amino acids. The hydrophobic residues were 38 to 75%. The peptide hydrophobicity (*H*) ranged from −0.08 to −0.94 and the hydrophobic moment (μH), which represents their amphipathicity [20], was 3.56 to 7.88.

The helical wheel (Edmundson wheel) was used to visually represent the amphipathic helix structures, wherein the amino acids are plotted in a rotating manner with rotation angle between consecutive amino acids of 100°. Thus, the final plot reveals whether hydrophobic amino acids are concentrated on one side and the polar or hydrophilic ones on the other side of the helix [21]. The predicted helical wheel structures are shown in Figure S1.

The peptides were then evaluated for biocidal activities on bacterial, fungal and mammalian cells, and stability in the presence of proteases and salt.

### 2.1. Effects of the Altered Physico-Chemical Parameters on the Antimicrobial Activity and Stability of the Designed Peptides

The PuroA peptide designed based on the “wild type” TRD of PINA exhibited potent antimicrobial activity (Table 2), as we had reported earlier [14], and its minimum inhibitory concentrations (MICs) were used for comparisons to those of other peptides. The peptides were tested together for antimicrobial activity against the Gram-positive *Staphylococcus aureus*, and the Gram-negative *Escherichia coli* and *Pseudomonas aeruginosa* bacteria. Selected peptides were also tested against two clinical isolates of methicillin-resistant *Staphylococcus aureus* (MRSA), M173525 and M180920. The minimum inhibitory concentrations (MICs), defined as the lowest peptide concentration that completely inhibited bacterial growth [22], were used for a comparative assessment of the antimicrobial activity of the different peptides. The backbone-cyclized variant (cyclic PuroA) showed a substantial decrease in activity, its MICs being four to eight-fold higher against all bacteria and showing a two-fold decrease against *C. albicans,* compared to the linear PuroA.

Dimerization considerably decreased the antibacterial potency of Di-PuroA but not its antifungal activity. Increasing the cationic charge was found to reduce the potency of R8, to four-fold less potent against *E. coli* and *S. aureus* and two-fold less potent against *C. albicans* than PuroA, but it should be noted that its hydrophobicity was also reduced. The peptides P1, with six Trp residues (one more Trp compared to PuroA), and dP1 (with several D-residues), showed two to four-fold greater potency against *C. albicans* and *P. aeruginosa,* respectively, but comparable potency to PuroA against *E. coli* and *S. aureus*. The PuroA-OH peptide displayed two-fold lower potency against all microorganisms compared to C-terminally amidated PuroA, confirming the significance of this amidation. Six of the short peptides (R7, W7, dW7, W8, WW, dWW) were generally more potent than PuroA against *E. coli* and *S. aureus*. Compared to PuroA, the shortest peptide R6 showed a two-fold reduction in potency against *E. coli* but, it was unchanged against *S. aureus* and interestingly, showed a two-fold enhancement in potency against *P. aeruginosa and C. albicans.* In addition, the antimicrobial activity of all L-amino acid peptides was similar to that of their counterpart diastereomers with 35–40% D-amino acids (Table 2). The activities of P1, W7 and WW against the two MRSA clinical isolates were greater than the activity of PuroA.

**Figure 1:**
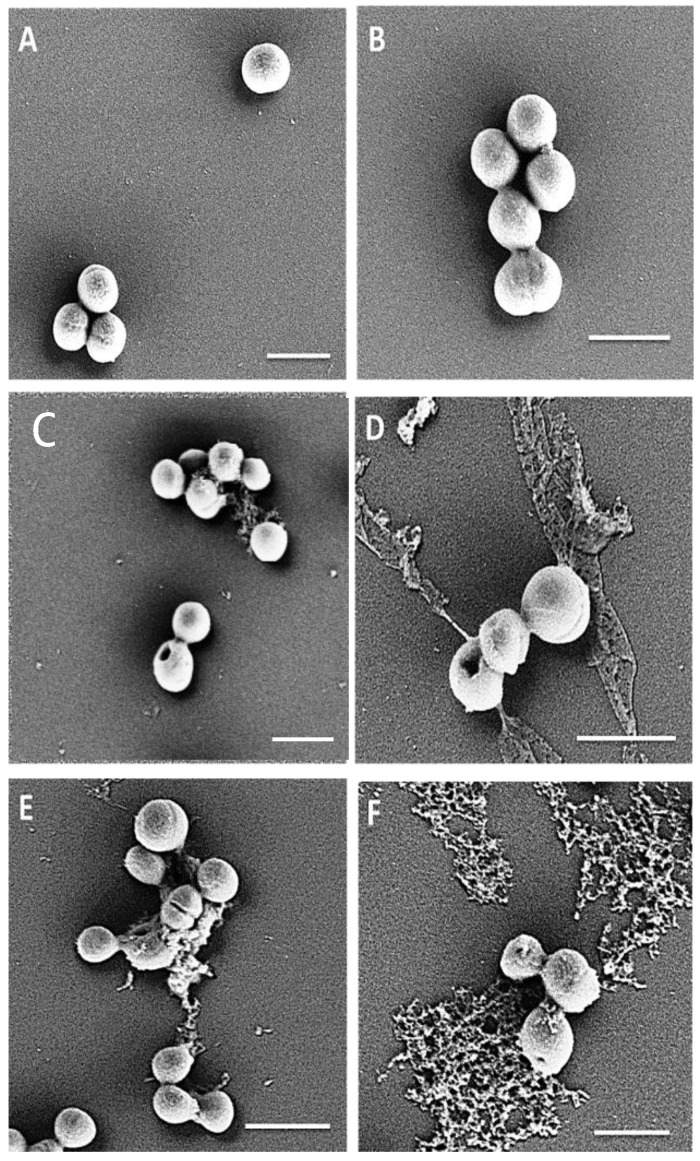
Scanning electron micrographs of MRSA M173525 planktonic cells treated with peptides at 1 × minimum inhibitory concentrations (MIC). (**A**) and (**B**): no-peptide control; (**C**): cells treated with P1 (16 μg/mL); (**D**): cells treated with PuroA (16 μg/mL); (**E**): cells treated with WW (4 μg\mL); (**F**): cells treated with W7 (8 μg\mL). Magnifications 40,000×, scale bar 1 µm.

A scanning electron microscope (SEM) was utilized to visualize the effects of peptides on the morphology of MRSA planktonic cells. Exposure of MRSA M173525 planktonic cells to PuroA, P1, WW and W7 peptides at their 1 × MIC resulted in visible damage, with pits or large pores observed on the surface of some cells and/or cellular material leakage, while untreated cells showed a regular shape with intact cell membrane (Figure 1). These are likely related to a carpet model type collapse of a large portion of the membrane.

For effective use of AMPs in clinical pharmacotherapy, they need to remain active under physiological levels of salt and actions of peptidases. NaCl is the predominant salt in vivo, with concentrations of 120–150 mM [23]. Hence, the MICs of peptides for *E. coli* and *S. aureus* were determined under different NaCl concentrations (Supplementary data Table S1). PuroA only tolerated 50 mM of NaCl and showed a two to four-fold increase in the MICs towards both bacteria in the presence of 100 and 150 mM NaCl (Table 3). Cyclic, dimeric and non-amidated forms were not active even at 50 mM NaCl. On the other hand, peptides P1, dP1 and R8 showed unchanged activity up to 150 mM NaCl. Among the short peptides, only the activities of R6 and R7 were affected and all others showed greater stability. Trypsin cleaves peptides on the C-terminal side of Lys and Arg side chains. Proteinase K is an endopeptidase that preferentially cleaves the peptide bond adjacent to the carboxyl group of aliphatic and aromatic residues with blocked alpha-amino groups [24]. All L-amino acid peptides seemed to have completely lost their activity after exposure to both proteases. In contrast, all diastereomers, i.e., dP1, dW7 and dWW, maintained full activity and exhibited strong resistance to protease degradation.

### 2.2. Effects on Biofilm Formation of the Clinical MRSA Isolates

To determine whether the peptides found to be effective on MRSA (Table 2) could be used as potential anti-biofilm agents to prophylactically prevent colonization, we evaluated their effects at sub-inhibitory concentrations (0.5 × MIC), inhibitory concentrations (1 × MIC) and super-inhibitory concentrations (2 × MIC) on both the initial cell adhesion by planktonic cells and preformed (6 h) biofilms of MRSA on polystyrene. The MIC defines the lowest peptide concentration that completely inhibits the bacterial growth [22], but it does not necessarily represent the minimum bactericidal concentration. Hence, peptide concentrations equivalent of 2 × MIC were used. The effects were assessed using two separate assays, crystal violet (CV) and methylthiazolyldiphenyl-tetrazolium (MTT) assays. The CV assay is used as an indicator of attached living and nonliving biomass in a biofilm, but does not represent the metabolic status of the cells, as it stains both attached viable and non-viable cells. Hence, the MTT assay, which is a respiratory indicator of live cells only, is used to complement it. MTT is reduced to a purple product in metabolically active cells, and can be measured calorimetrically. The CV assays showed that the adhesion of MRSA cells to polystyrene was significantly affected, regardless of PuroA and P1 peptide concentrations (Table 4). The percentage inhibition of initial cell attachment did not vary noticeably from 0.5 × MIC to 2 × MIC. PuroA at 0.5 × MIC reduced the biomass attachment by about 70%, and penetrated the biofilms and killed 92% of the cells, as assessed by MTT assay, and these were 79% and 95%, respectively, at 2 × MIC. All peptides also significantly reduced the biomass and viability of the preformed 6h biofilms. The inhibition of biofilm formation was confirmed by confocal microscopy, wherein the peptides remarkably reduced cell attachment to the surface of the glass slide of a static chamber, and any attached cells appeared dead (Figure S3).

### 2.3. Prediction of 3D Structures and Determination of Peptide Conformations

**Figure 2:**
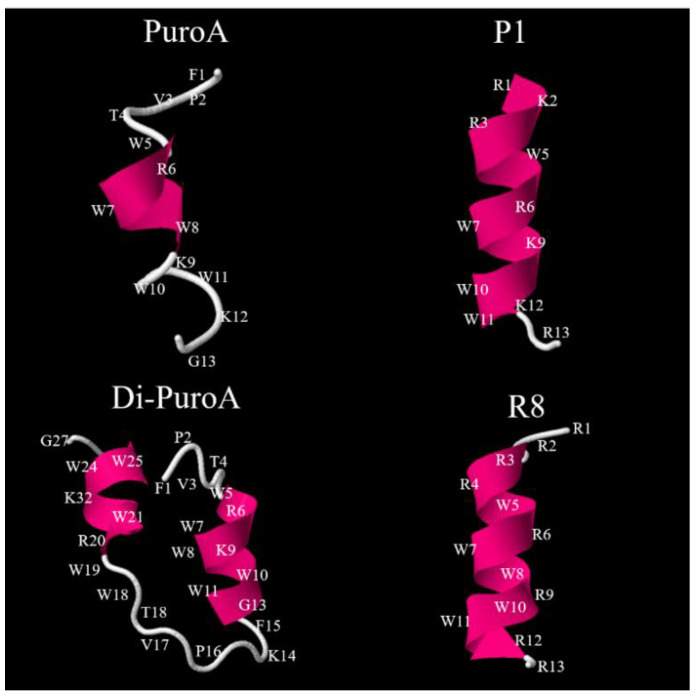
Three-dimensional structure projections of peptides. The structures were predicted by I-TASSER (http://zhanglab.ccmb.med. umich.edu/I-TASSER).

The 3D structure projections of the longer peptides (≥13 amino acids) including PuroA, Di-PuroA, P1 and R8 were conducted using homology modelling by I-TASSER (https://zhanglab.ccmb.med.umich.edu/I-TASSER/), noting that the 3D structures of short (≤8 amino acids) peptides cannot be predicted with it. PuroA was found to have a helical segment spanning Trp5 to Trp8, while P1 and R8 had longer helical segments, from Arg1 to Lys12 and from Arg3 to Arg 12, respectively (Figure 2). On the other hand, Di-PuroA contains two helical segments, from Trp5 to Gly13 and from Arg20 to Trp25.

**Figure 3:**
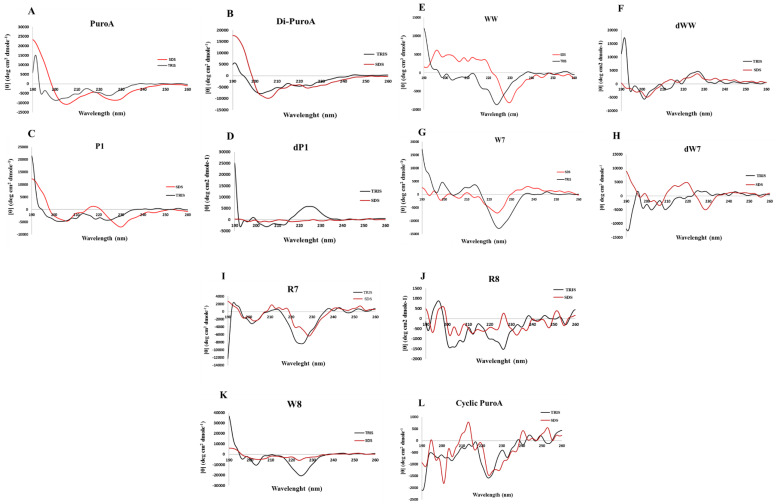
Circular dichroiism spectra of peptides. All spectra were recorded in 20 mM Tris buffer (black lines) or 20 mM SDS (red lines). θ = mean residue molar ellipticity.

For peptide conformation determinations, the circular dichroism (CD) spectra of peptides were measured in 20 mM Tris-HCl buffer (pH 7.4) (mimicking aqueous environment) and 20 mM SDS detergent (mimicking negatively charged cell membrane environment). For a random structure, there is only one minimum peak at around 200 nm. However, all spectra obtained showed no typical random coil in 20mM Tris-HCl. The CD spectra in 20 mM SDS showed double minima for PuroA, Di-PuroA and P1 (Figure 3A–C). However, these shifted to longer wavelengths relative to the 208 nm and 222 nm, typically obtained for an α-helix. This shift can be attributed to the high content of Trp, as observed for other well-studied Trp-rich peptides, indolicidin [25] and tritrpticin [26]. The CD spectrum of PuroA is consistent with that of Jing et al. [13]. R8, cyclic PuroA, W7, W8, WW and R7 seem unlikely to adopt ordered secondary structures in SDS. There is a limitation of CD spectroscopy for secondary structure analysis for peptides <10 residues. The short peptides W7 and WW may not adopt any particular secondary structure for their potent antimicrobial activity. In addition, CD measurement is based on molecular chirality, hence mixed L- and D-amino acids in a peptide may null its chirality, leading to no difference between the left- and right-circular polarized light. Substitutions of D-amino acids in dP1, dWW and dW7 led to significantly different spectra in both environments (Figure 3D,F,H).

### 2.4. Potential DNA-Binding Ability and Intracellular Action

In order to investigate whether the peptides may have a possible intracellular mechanism of action, their ability to bind to DNA in vitro was indirectly investigated using a gel retardation assay. The effect of different peptide concentrations on the migration of the plasmid DNA in agarose gels was monitored. Representative results are shown in supplementary data Figure S2. PuroA and PuroA-OH (not shown) both completely inhibited the migration of DNA at 32 µg/mL. The cyclized PuroA was shown to be a weaker DNA binder (migration inhibition at 64 μg/mL), while Di-PuroA was shown to be a stronger binder (inhibition at 8 μg/mL). Interestingly, this difference seemed unrelated to their low antibacterial activity (Table 2), suggesting their mechanisms of action may be different. In contrast, P1 (Figure S2) and dP1 (not shown) showed the strongest ability to inhibit plasmid migration (at 8 μg/mL) but also strong antimicrobial activity. The short peptides WW and dWW presented lower binding (inhibition at 32 μg/mL) than the longer P1 and dP1. R6 and R7 demonstrated the weakest abilities, with migration inhibition at 250 and 125 μg/mL, respectively.

**Figure 4:**
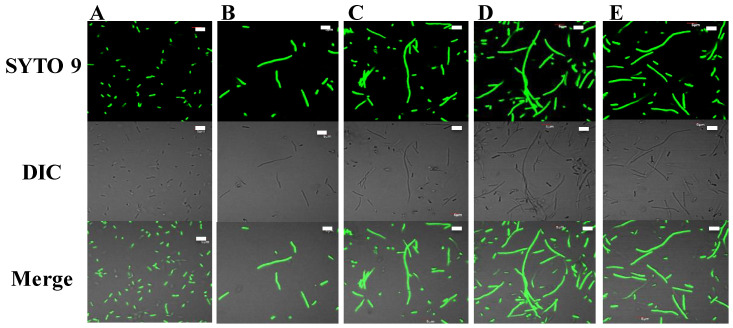
Filamentation in *E. coli* cells induced by peptides. Cells were observed using confocal microscopy at × 1000 magnification under oil immersion, after incubation with peptides for 3 h at 37 °C. (**A**): no-peptide control; (**B**–**E**): cells treated with varying final concentrations of peptides as per their 1 × MIC; (**B**): PuroA (16 µg/mL); (**C**): W7 (4 µg/mL); (**D**): P1 (16 µg/mL); (**E**): WW (8 µg/mL). Scale bar 5 µm. DIC: Differential interference contrast.

Filamentation is a defect wherein a bacterial cell continues to grow without septum formation, possibly due to intracellular events such as blocking of DNA synthesis [27,28] or inhibition of membrane proteins [29] including a FtsZ, a bacterial tubulin with functions in the divisomes of Gram-positive and Gram-negative bacteria [30]. It can thus be taken as an indicator of mechanism(s) of action of a peptide not directly involving to membrane disruption. Therefore, an *E. coli* filamentation assay was used as an indicator of whether the peptides potentially affected these processes. After incubating *E. coli* cells with peptides at MIC for 3 h, the cells were stained with SYTO 9 (a green florescent cell-permeant nucleic acid dye) and inspected for filamentous growth of cells (due to potential defects in cell division machinery) using confocal microscopy and differential phase contrast microscopy. All peptides except dimeric PuroA induced filamentation at their MIC, with cell lengths increasing about five-fold compared to normal (Figure 4).

### 2.5. Haemolytic Activity and Cytotoxicity Against Mammalian Cells

**Figure 5:**
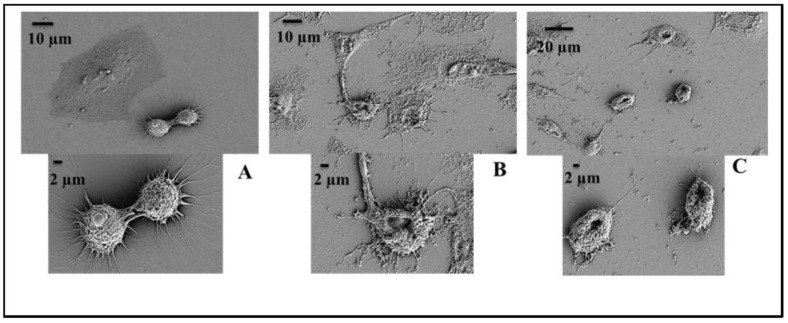
Scanning electron micrographs of HeLa cells after treatment with P1 and Di-PuroA peptides. (**A**): no-peptide control; (**B**): P1 (32 µg/mL); (**C**): Di-PuroA 64 µg/mL.

As a primary characterization of the cytotoxic properties of the designed TRPs, their haemolytic activities against sheep red blood cells (RBCs) were measured. The haemolytic activities of the peptides were determined by measuring the amount of haemoglobin released from sheep RBCs suspension after exposure to various peptide concentrations for 1 h. Within detectable limits (16–500 µg/mL), all peptides were non-toxic to sheep erythrocytes. Many TRP have been reported to show antitumor activity [31,32], hence the MTT assay was used to test any in vitro cytotoxicity of the peptides to two mammalian cell lines; mouse fibroblast NIH-3T3 cells and the human cervical carcinoma HeLa cells. PuroA, Di-PuroA, P1, dP1, R6, R7, W7 and dWW showed low cytotoxicity to NIH-3T3 mouse fibroblasts, with IC_50_ (peptide concentration that causes 50% growth inhibition) of 250 µg/mL, and no cytotoxicity for cyclic PuroA, PuroA-OH, R8, dW7, W8 and WW (Table 5). The trend of activity towards the human cervical carcinoma HeLa cells was not as clear as that towards microbial cells but, in general, the peptides were less effective against HeLa cells. R8, dP1, W7, dW7, W8, WW and dWW showed low cytotoxicity (IC_50_ 125–250 µg/mL). The therapeutic index (TI) is a widely used parameter to represent cell selectivity and specificity of peptides. It is commonly calculated by the ratio of HD50 (peptide concentration that lyses 50% of the RBCs) and GM (geometric mean of MICs against tested microorganisms), or the ratio of IC_50_ normal (peptide concentration that causes 50% growth inhibition in normal mammalian cell line) and IC_50_ cancer for cancerous cells [33,34]. Most notably, P1 and Di-PuroA displayed relatively strong cytotoxicity and specificity, both with an IC_50_ of 32 µg/mL and therapeutic index of 7.8. SEM showed that most of the P1-treated HeLa cells were damaged significantly (Figure 5), while the untreated cells showed normal morphology of flattened or rounded shape with several microvilli and extending lamellipodia.

## 3. Discussion

AMPs with broad-spectrum antimicrobial activity, metabolic stability and low cytotoxicity to normal mammalian cells are being researched actively for controlling persistent and drug resistant infections. Naturally occurring peptides with these attributes are rare; hence, rational design based on a thorough understanding of peptide structure–functionality can fast-track their development. Several structure–activity relationship studies of Trp- and Arg-rich AMPs, such as lactoferricin [16] and indolicidin [17] have indicated that conserved RW motifs are relevant to the antimicrobial functionality. By using the Trp-rich sequence of PuroA as a template, the current work aimed to identify factors that can lead to better potency and cell selectivity. Peptides were designed by varying certain physico-chemical parameters that are known to contribute to the biocidal activities of AMPs, i.e., net charge, length, primary structure, hydrophobicity and amphipathicity. Their activities were then tested on bacterial, Candida and mammalian cells. The outcomes are discussed below.

The cationic character of the peptides is reported to affect the initial interaction of peptides with microbial and/or cancer cell membranes, consequently affecting selective toxicity [35]. Hence, to study the effects of cationic charge, the predicted net charge of PuroA was increased by substituting all non-Trp residues with cationic ones. Trp and Arg complement the biochemical properties of each other in many Trp-Arg rich AMPs [6]. The Arg side chain has a more dispersed positive charge compared to Lys, and contributes to the “snorkeling behaviour” of Arg-rich AMPs [36]. Hence, Arg was chosen to create R8 (net charge +8); however, it showed reduced antibacterial and antifungal activity. This may be due to its strong interaction with the negatively charged phospholipid headgroups in cell membranes, preventing conformational change and consequently the kinetics of membrane permeabilization and translocation [7]. On the other hand, replacing the uncharged residues of PuroA with Arg and Lys in P1 (+7) improved the antimicrobial and anti-HeLa cell activity. The results confirm that increasing the net charge beyond +7 does not necessarily improve antimicrobial activity [7]. Unlike other studies [37], increasing the net charge to +7 or +8 did not increase the haemolytic activity of P1 or R8. However, as expected, the high net positive charge may have overcome the charge-shielding effect of salt (salt ions competing and reducing the electrostatic attraction between cationic AMPs and negatively charged microbial membranes) [38].

In light of the well-reported direct correlation of high cationic charge to antimicrobial activity and salt stability [38], the high potency and salt stability of the shorter Trp-rich peptides (net charge +2 or +3; similar to PuroA) was unexpected. The amphipathic structure and cluster of Trps may protect them from salt inactivation effects. Increasing Trp content had improved the antimicrobial activity and salt tolerance of the decamers in the D0 peptide (SGKLCCRRKK-NH_2_) [38]. Based on this and our results, adding Trp residue(s) could be a useful approach to designing AMPs effective at physiological salt concentrations, for in vivo use. C-terminal amidation is a common post-translational modification of defence peptides [39], suggesting its biological relevance. Dennison et al. [40] suggest that the C-terminal amide moiety has a variable effect on the activity and/or selective toxicity of AMPs on both microbial and cancer cells. Our results support this partially, as the C-terminally amidated PuroA had higher antimicrobial activity and slightly increased cytotoxicity to mouse fibroblast cells compared to PuroA-OH but exhibited negligible changes to anti-HeLa cell activity.

The length of AMPs is a very important factor in their activity [41], toxicity [42] and mode of action [43]. Due to the high cost of production of synthetic peptides, short peptide candidates are desirable. Hence an effort was made to further identify the active center of PuroA, the TRD being the focus due to its proven antimicrobial activity [14]. The short linear peptides, R6, R7, W7, W8 and WW, showed greater activity than PuroA against the bacteria and yeast, insignificant cytotoxicity towards sheep RBCs and mouse fibroblasts, and excellent salt stability. These data will help in advancing affordable AMP design for large scale production. However, the effects of peptide length on biofilm inhibition are unclear and so this needs further work. Although W7 and WW were highly effective against the planktonic cells of MRSA, they showed similar inhibition effects to PuroA on the initial attachment of cells and eradication of preformed biofilms. Interestingly, length did seem to play a role in anticancer activity, as WW (shortest form of P1) showed reduced effects on HeLa cells. Previous studies have stated no clear correlation between the length and toxicity or selectivity of anticancer peptides [35,44]; hence, this factor needs to be investigated further.

Hydrophobicity is considered to be independent of other physicochemical characteristics of AMPs [7]. It is reported to greatly affect the AMP activity, range of targets and cytotoxic selectivity, as it strongly controls partitioning of the peptide into the membrane hydrophobic core [45]. However, we found no clear correlation between hydrophobicity and antimicrobial activity; e.g., PuroA and WW have similar hydrophobicity, but WW had greater antimicrobial potency. In addition, these peptides were the most hydrophobic (Table 1, *H* = −0.08), but did not show greater cytotoxicity against sheep RBCs or mouse fibroblast cells. It was also difficult to establish a correlation between hydrophobicity and anticancer activity, as P1 and dimeric PuroA had very different hydrophobicity but similar toxicity to HeLa cells (Table 5).

Partitioning of AMPs into membranes is suggested to be mainly accompanied by secondary structure formation [46]. AMPs can adopt either α-helical or β-sheet structures, the degree of structuring affecting their activity [4]. In this study, using CD spectroscopy, PuroA was found to be unstructured in aqueous buffer, as were the other designed TRPs, as expected for linear peptides of ≤14 residues. In the presence of detergent, only PuroA, Di-PuroA and P1 formed a more ordered structure and the other peptides did not adopt any well-defined α-helical or β-sheet structures. Interestingly, they showed considerably different antimicrobial activities (Table 2), while the short diastereomeric peptides—less likely to form a secondary structure—showed potent activities. Of the archetypal TRPs, only lactoferricin (LfcinB) adopts an amphipathic β-sheet structure [47], while indolicidin [48] and tritrpticin [26] adopt stable amphipathic structures in SDS micelles. Jing et al. [13] also showed that PuroA forms a well-defined amphipathic conformation when bound to SDS micelle. Our results lead us to agree with Rozek et al. [48], that AMPs do not need to conform to a recognized secondary structure to exert antimicrobial effects; adopting an amphipathic conformation seems more important.

Proteolytic degradation is a significant obstacle for the clinical use of AMPs, hence several strategies have been used to decrease their susceptibility to serum proteases and increase their half-life, e.g., cyclization [49], or introduction of the unnatural D-amino acids [19,50]. The incorporation of D-amino acids is known to affect the helicity, hydrophobicity and selectivity of peptides [51,52]. Hence, we designed diastereomers with 35–40% D-amino acids spaced with small stretches of L-amino acids. Most interestingly, we found that, irrespective of the sequence, helicity or hydrophobicity, the antimicrobial activity of all L-amino acid peptides was similar to that of the corresponding diastereomers, and all L-isomers were fully inactivated by trypsin and proteinase K, while the diastereomers retained their activity for up to 3 h. In terms of cell selectivity, in contrast to previous studies [51], diastereomeric peptides did not exhibit greater selectivity towards mammalian RBCs or fibroblasts compared to L-isomers. In fact, dP1 showed similar and dWW showed less selectivity than the L-isomers. These findings are noteworthy in that they suggest that once a potent peptide is designed, D-amino acids may be introduced at specific positions to produce one with the same potency and host cell spectrum but reduced susceptibility to enzymatic degradation.

The strategy of cyclization of the AMP backbone has led to potent antimicrobial analogs of indolicidin [49] and tritrpticin [53], with decreased haemolytic activity and/or increased proteolytic stability [49,53]. There are a number of naturally-occurring cyclic AMPs, like microcin J25 [54]. In this study, a cyclic analog of PuroA with an additional head-to-tail bond in the backbone was designed and synthesized. Interestingly, it exhibited a decrease in both the activity against bacteria and fungi and cytotoxicity against NIH-3T3 fibroblasts, but had the same haemolytic activity as the linear PuroA. Nguyen et al. [53] reported a similar negative effect of cyclization on the antibacterial activity of tritrpticin; however, a different cyclization strategy was used, through a disulfide bond between Cys residues. The role of cyclization in biological function and mode of action of AMPs thus needs to be better understood.

The aggregation or oligomerization of AMPs before or at binding to the cell membrane is important for pore formation [55]. Additionally, host cell selectivity of AMPs is affected by self-association (oligomerization) in aqueous environments [56]. Thus, some dimeric peptides have been designed and synthesized, however, some showed improved pharmaco-technical characteristics of AMPs (physical characteristics such as hardness, solubility and disintegration time) [57], while others showed reduced antimicrobial activity and/or increased cytotoxicity [58]. In our study, the C-terminal dimeric analog (PuroA)_2_k did not exhibit improved antimicrobial activity, salt or protease resistance, or induction of *E. coli* cell filamentation, but promoted aggregation of the bacterial cells. This is similar to the dimeric aurein 1.2 inducing aggregation of *C. albicans* cells [58]. AMPs are reported to selectively interact with lipopolysaccharides in Gram-negative bacteria [59]. Hence aggregation of *E. coli* cells and reduced antibacterial activity could be due to interaction with the cell wall, preventing the dimeric peptides from reaching the cell membrane. Its ability to induce aggregation also makes it an attractive candidate to inhibit adhesion of bacteria to biological and medical surfaces. Dimeric AMPs have also shown selective anti-cancerous activity [60]. Our study supports this, as the dimeric PuroA showed selective cytotoxicity to HeLa cells and reduced their viability, but no significant cytotoxicity to NIH-3T3 fibroblasts or RBCs. Dimerization thus seems to be a useful strategy to design peptides with anticancer activity. 

Preliminary investigations of the ability of peptides to bind DNA were undertaken using in vitro (gel retardation) and in vivo *E. coli* cell filamentation assays. The designed peptides showed different affinities for the plasmid DNA in vitro. However, these were not correlated to their antimicrobial activities; e.g., Di-PuroA and P1 showed the strongest affinity for plasmid DNA (Figure S1), though Di-PuroA was much less active against bacteria and yeast than P1. Hsu et al. [61] proposed that indolicidin could insert into DNA, its tryptophans stacking between the bases or sugars, and its cationic residues potentially binding electrostatically with phosphate groups. Substituting Trp 3 or 4 in indolicidin with Lys resulted in stronger DNA binding [62], and substituting the Trp–Trp at the central Pro-Trp-Trp-Pro (PWWP) motif considerably decreased its ability to bind and stabilize duplex DNA [63]. In our results, the number of Trp compounds residues is possibly relevant to the strong bonding of Di-PuroA and P1, as both have ≥ 6 Trp. However, no correlation was evident with net charge, as R8 (+8) showed less affinity to plasmid DNA than W8 (+3) (Figure S1).

Filamentation is a defect in cell division where the cell continues to grow without septum formation, and is suggested to occur due to blocking of DNA synthesis [27,28] or inhibition of relevant membrane proteins [29]. Recent research on a natural peptide Temporin L, which causes filamentation of *E. coli* cells, shows a GTPase called FtsZ, a bacterial tubulin homolog with functions in the divisome of Gram-positive and Gram-negative bacteria, to be a target for this peptide [30]. It can thus be taken an indicator of mechanism(s) of action of a peptide not related to membrane disruption. Trp-rich peptides such as indolicidin [27] and LfcinB [64] are known to induce filamentation of *E. coli*. All the peptides designed in our study except the dimeric PuroA induced filamentation in *E. coli* cells at their MIC, regardless of their physicochemical characteristics, suggesting a role for Trp residues and potential intracellular mechanisms.

Unlike most antibiotics which have defined targets in microbial cells, the mechanisms of action of AMPs are diverse and complex, involving various mechanisms of disruption of cell membranes and/or binding to and affecting multiple intracellular structures or biomolecules. Our earlier studies showed that *Saccharomyces cerevisiae* cells treated with PuroA appeared intact, with pore-like structures appearing in the membranes; hence, it was suggested to exert its antimicrobial effects by disrupting the membrane integrity followed by intracellular activity [65]. We next applied time-lapse fluorescence lifetime imaging microscopy (FLIM) and time-lapse fluorescence microscopy, to directly observe the localization and interaction kinetics of fluorescently-tagged PuroA on single cells of *Candida albicans* in real time. This study showed a dual mechanism, with the disruption of the membrane integrity possibly being the main cause of cell death, but only after peptide translocation and binding to intracellular targets [18]. The observations in the current work, of induction of filamentation in *E. coli* by PuroA (and other peptides)*,* and the large pits/holes seen in MRSA cells, suggests that PuroA and perhaps other peptides may be exerting their bactericidal effect using similar mechanisms, i.e., translocation across the membrane, binding to nucleic acids and other targets (including some related to cell division), accumulation at the cell membrane and eventually disrupting it at a specific threshold, possibly by pore or carpet mechanisms. Further investigations will be aimed at deciphering the cellular targets, the sequence of events during biocidal attacks on bacterial or fungal cells, as well as development of any host cell resistance, which may also be specific to the host species or the peptide.

## 4. Materials and Methods

### 4.1. Peptide Design

The peptide PuroA (FPVTWRWWKWWKG-NH_2_) designed based on the TRD of wheat seed protein PINA, encoded by the “wild type” allele *Pina-D1a* [11] (Genbank DQ363911) and 13 other peptides using its sequence as framework were designed (Table 1). The variations (detailed in Section 2) included substitution of non-Trp residues with Arg (peptide R8), substituting all uncharged residues with Arg and Lys and insertion of an additional Trp (P1), short peptides retaining the Arg, Lys and Trp (R6, R7, W7, W8, WW), a cyclic analog, a dimeric analog (PuroA)_2_K, diastereomeric analogs (dP1, dW7, dWW) with D-amino acids at positions which allow short consecutive sections of one or two L-amino acids that cannot adopt α-helices [19], and C terminal amidation of all peptides except PuroA-OH. All peptides were commercially purchased from Biomatik Corporation (Kitchener, Ontario, CA) or Mimotopes, (Clayton, Victoria, Australia) at >95% purity. Stock solutions (1 mg/mL) of peptides to be used in antimicrobial assays were prepared in 0.01% glacial acetic [22], those of peptides to be used in haemolytic and cell culture assays were prepared in sterile MilliQ water, and all stored at −20 °C.

### 4.2. Prediction of the Physicochemical Properties of Peptides

The net charge was calculated as sum of the number of positively-charged amino acids (Arg, Lys), as no peptide had the negatively charged amino acids (Asp, Glu). The C-terminal amidation was not included in the calculation for consistency with the published reports [15]. The isoelectic point (pI) and molecular weight were predicted using the “compute pI/MW Tool” in the Expert Protein Analysis System (ExPAsy) (http://au.expasy.org/tools/pi_tool.html) (The SIB Swiss Institute of Bioinformatics, Lausanne, Switzerland). Charge density (ChD) was calculated by dividing the net charge by the chain length of the peptide. Mean values of hydrophobicity (H) were calculated using the consensus values of hydrophobicity scale [66]. Hydrophobic ratio (HR) expresses the percentage of hydrophobic amino acid (Val, Phe, Thr, Trp). The “GRAVY” (grand average of hydropathy) was calculated by dividing the sum of hydropathy values of all amino acids by peptide length. The hydrophobic moment (µH), representing peptide amphipathicity [20], and the helical wheel projections (Figure S1) were calculated using Helical Wheel Projections (http://rzlab.ucr.edu/scripts/wheel/wheel.cgi) (Riverside, CA, USA).

### 4.3. Antibacterial Activity and Salt Stability Assays

The activity of peptides was determined against the Gram-positive *Staphylococcus aureus* (ATCC 25923), and the Gram-negative *Escherichia coli* (ATCC 25922) and *Pseudomonas aeruginosa* (ATCC 9027) bacteria. Selected peptides were also tested against two clinical isolates of methicillin-resistant *Staphylococcus aureus* (MRSA), M173525 and M180920, kindly provided by Dr P. B. Ward (Microbiology Department, Austin and Repatriation Medical Centre, Heidelberg West, Victoria, Australia). The Minimum inhibitory concentration (MIC) of peptides was determined by the microtitre broth dilution method, as described in Phillips et al. [14]. Briefly, bacteria were grown in Mueller-Hinton Broth (MHB) at 37 °C overnight with shaking (200 rpm). The cultures were adjusted to 0.5 McFarland turbidity standard (approximately 1 × 10^8^ colony forming units (CFU)/mL), then diluted 1:100 in MHB (1 × 10^6^ CFU/mL). A total of 75 μL of these suspensions were mixed with 25 μL of peptide solutions (final concentrations 250 to 0.5 μg/mL) and the microtitre plates incubated overnight at 37 °C. Positive growth controls (inoculum and MHB, with no peptide) and sterility controls (0.01% glacial acetic) were also included. Post-incubations, the absorbance of cultures at 595 nm was measured using a microplate reader (POLARstar Omega, Germany). The MIC was defined as the lowest peptide concentration that completely inhibited bacterial growth [22]. To evaluate salt sensitivity of peptides up to physiological levels (150 mM NaCl), overnight cultures of *S. aureus* and *E. coli* adjusted to 0.5 McFarland turbidity standard were diluted 1:100 in MHB containing 0, 50, 100 or 150 mM NaCl, and the MICs of peptides determined as above. All tests were conducted in triplicate. The results are shown as the mode of values obtained.

### 4.4. Anti-Candida Activity Assay

The assay was carried out as per Alfred et al. [65]. *Candida albicans* (FRR 5580) was grown in potato dextrose broth (PDB; Difco-BD, Bergen County, NJ, USA) overnight at 30 °C with shaking (200 rpm) until exponential phase, then adjusted to 0.5 McFarland turbidity standard (approximately 1 × 10^6^ cells/mL) and diluted 1:200 with PDB to 0.5 − 2 × 10^3^ cells/mL. Peptides in 25 μL volumes were mixed with 75 μL of the yeast suspensions. The controls were included as above. The plates were incubated overnight and absorbance was recorded. All the tests were conducted in triplicate.

### 4.5. Proteases Stability Assay

The effect of two well-studied proteases, porcine trypsin and proteinase K from *Tritirachium album*, on the activity of peptides was investigated as per Carmona et al. [50]. Briefly, 1000 µg of a peptide was incubated with 50 µg of a protease (20:1 *w/w*) at 37 °C for 3 h in phosphate buffered saline PBS (pH 7.4). The enzyme activity was then terminated by heating (70 °C, 5 min) and the samples centrifuged at 12,000× *g*. The activity of the supernatant on *E. coli* and *S. aureus* was tested as above, in triplicate.

### 4.6. Three Dimensional Structure Prediction

The 3D structure of the long peptides (≤13 amino acids, Table 1) was predicted using the iterative threading assembly refinement (I-TASSER) server (https://zhanglab.ccmb.med.umich.edu/I-TASSER/) (Ann Arbor, MI, USA). The structures were constructed from multiple threading alignments of amino acid sequences and iterative structural assembly simulations [67]. The ab initio approach was used for structure prediction due to unavailability of an appropriate template, and the search returned proteins segments with close structural similarity to the peptides.

### 4.7. Circular Dichroism Spectroscopy

CD spectra of the peptides were measured with a Jasco J-815 spectrophotometer (Japan Spectroscopic Company, Tokyo, Japan), as per Jing et al. [13]. Each spectrum (190–260 nm) was the average of three scans, obtained by using a quartz cuvette with a 1 mm path length at 25 °C. The measurements were performed in continuous scanning mode with scanning speed of 50 nm/min, step size of 0.1 nm, 1.0 nm bandwidth and a 4 s response time of the detector. The samples contained 40 µM of peptide in 20 mM Tris-HCl buffer (pH 7.4) or 20 mM sodium dodecyl sulphate (SDS) detergent. The spectra were baseline-corrected by subtracting a blank spectrum containing all components except the peptide. After noise correction, ellipticities were converted to mean residue molar ellipticities [θ] expressed as degree square centimetre per decimole (deg cm^2^/dmol).

### 4.8. In Vitro DNA-Binding Assay

The ability of peptides to bind to purified DNA (indicative of in vivo DNA binding potential) was tested by the modified gel retardation assay developed by Alfred et al. [65]. The DNA of pBlueScript SK+ (100 ng) was incubated with peptides (to final concentrations of 8, 16, 32, 64, 125, 250 or 500 µg/mL) in 15 µL volumes made with sterile MilliQ water, the held at room temperature for 1h. Loading dye was added to each sample and the samples electrophoresed on 1% agarose gels. The gels were then immersed in ethidium bromide (0.5 mg/mL) then visualized and imaged

### 4.9. E. coli Filamentation Assay

The ability of peptides to induce filamentation in *E. coli* was assessed as per Alfred et al. [65] and Subbalakshmi and Sitaram [27]. *E. coli* was grown to logarithmic phase (OD_600_ = 0.2) in MHB, then diluted to 1 × 10^8^ CFU/mL. Twenty five μL of peptide solution (final concentration 2–250 μg/mL) was incubated with 75 μL of the bacterial culture for 3 h at 37 °C. Cultures mixed with sterile MilliQ water or PuroA were used as negative and positive controls, respectively. The cells were then stained with SYTO 9 (green florescent cell-permeant nucleic acid dye for living cells) (Molecular Probes^®^, Eugene, OR, USA) and visualized using an inverted confocal microscope (FluoView FV1000; Olympus, Notting Hill, Victoria, Australia), 100× oil immersion objective and a 488 nm argon laser for excitation of SYTO 9. The bandpass emission filter was U-MNIBA2 for the 488 nm green channel and no bandpass filter for differential interference contrast (DIC). All filters were cycled with a BX filter wheel.

### 4.10. Testing the Activity of Peptides on Biofilms of Clinical MRSA Isolate M173525

The effects of PuroA and other selected peptides (based on their antibacterial activity) on both the initial cell attachment and 6 h preformed biofilms of MRSA on polystyrene were tested as described by Jadhav et al. [68], at their respective 0.5 × MIC, 1 × MIC, and 2 × MIC concentrations. All tests were conducted in triplicate. For testing the inhibition of initial cell attachment, 100 μL of peptide solutions were added to the microtitre plates, then mixed with 100 μL of M173525 cell suspension (1 × 10^6^ CFU/mL, cultured in tryptic soy broth medium (TSB; Oxoid) containing 1% glucose), or 100 μL of sterile MilliQ water as negative control. The plates were incubated at 37 °C for 24 h to allow cell attachment. The biofilm formation was then assessed using CV and MTT assays. For testing the inhibition of preformed biofilms, 100 μL of the M173525 suspension (1 × 10^6^ CFU/mL) was added to the wells of microtitre plates and incubated for 6 h at 37 °C to allow cell attachment and biofilm formation. Peptide solutions (100 μL) were then added, the plates incubated at 37 °C for 1 h and the biofilms assessed.

### 4.11. Biofilm Biomass Assay (Crystal Violet Assay)

The assay works as an indicator of attached living and non-living biomass [69]. Indirect assessment of cell attachment of MRSA was estimated using the modified CV assay as per Jadhav et al. [68]. After incubations of the plates (for 24 h or 1 h, as above), the culture medium from each well was removed gently and any non-adherent bacteria removed by washing the plates five times with sterile distilled water (dH_2_O). The plates were air-dried, then oven-dried at 60 °C for 1 h. The biofilm cells in the wells were stained with 100 μL of 1% CV and incubated for 15 min at room temperature. Excess stain was removed by washing the plates three times with sterile dH_2_O. Ethanol (95%, 125 µL) was transferred to each well to de-stain the cells. The de-staining solution from each well was then transferred to a new plate and its absorbance measured at 595 nm. The percentage inhibition of biomass formation concentration was calculated as: ((OD_595 nm_ control well without peptide − OD_5 95_ well with peptide)/OD_595 nm_ control well without peptide) × 100. All tests were conducted in triplicate.

### 4.12. Biofilm Metabolic Activity Assay (MTT Assay)

A modified methylthiazotetrazolium (MTT) (3-[4,5-dimethyl thiazol-2-yl]-2, 5-diphenyltetrazolium bromide) reduction assay [70] was used, wherein the blue product (MTT salt reduced to formazan by dehydrogenases) is taken as a respiratory indicator of live cells. Briefly, MTT (Sigma-Aldrich, Gillingham, UK) was dissolved in PBS to 5 mg/mL. After incubations of plates (for 24 h or 1 h, as above), the culture medium was gently removed and the plates air-dried. PBS (100 μL) and MTT (5 µL, 5 mg/mL) were added to each well and the plates incubated for 3 h at 37 °C. The insoluble purple product was dissolved in 100 µL of dimethyl sulphoxide (DMSO, Sigma-Aldrich, Gillingham, UK) and the absorbance measured at 570 nm. The % inhibition of metabolic activity was calculated as: ((OD_570 nm_ control well without peptide − OD_570 nm_ well with peptide)/OD_570 nm_ control well without peptide) × 100. All tests were conducted in triplicate.

### 4.13. Visualization of the Effect of Peptides on Initial Adhesion of MRSA M173525

The inhibitory activity of P1 peptide on initial adhesion and biofilm formation of MRSA isolate M173525 was visualized using confocal microscopy as described by Anunthawan et al. [71], with some modifications. Briefly, 500 µL of the peptide P1 at 1 × MIC were added to coverglass cell culture chambers (Nunc™ Lab-Tek™ II, Thermo Scientific) together with 500 µL of overnight cultures of MRSA M173525 (adjusted to 1 × 10^6^ CFU/mL), or sterile MilliQ water for negative control. The chambers were incubated at 37 °C for 24 h, then washed gently with PBS and the remaining biofilms stained with SYTO 9 and propidium iodide (PI) (a red fluorescent stain for dead cells) (Molecular Probes^®^, USA) for 15 min. They were then observed with an inverted confocal microscope as above, with excitation wavelengths of 488 and 568 nm used to detect SYTO 9 and PI, respectively. A 488 nm argon laser was used for excitation of SYTO 9 and a 543 nm helium neon laser was used for excitation of PI.

### 4.14. Haemolytic Activity

Hemolysis was assessed as per Dathe et al. [72]. Sheep red blood cells (RBC) were collected from whole defibrinated blood (Amyl Media, Australia) by centrifugation at 1200× *g* for 10 min at 4 °C, then washed three times with PBS, resuspended in 2 mL PBS (1.9 − 2.5 × 10^9^ cells/mL) and diluted 10% in PBS just before testing. Cell suspension (25 μL aliquots) were incubated with peptides (final concentrations 16 to 500 μg/mL) for 1h at 37 °C with shaking (160 rpm). The samples were then centrifuged at 3200× *g* for 5 min and absorbance of supernatants measured at 540 nm. PBS and 1% Triton X-100 (Sigma, St Louis, MO, USA) were used as negative (0%) and positive (100%) controls, respectively. Haemolytic dose (HD_50_) was defined as peptide concentration that lysed 50% of RBCs, determined as: ((OD_540 nm_ peptide − OD_540 nm_ PBS)/(OD_540 nm_ Triton − OD_540 nm_ PBS) × 100. All tests were carried out in triplicate.

### 4.15. Cytotoxicity Against Mammalian Cells

Cytotoxicity to human cervical carcinoma HeLa cells and mouse fibroblastic NIH-3T3 cells was determined using the MTT assay as per Scudiero et al. [73] with minor modifications. The cells were maintained in Dulbecco’s Modified Eagle Medium (DMEM) with 10% foetal bovine serum (FBS) and 1% penicillin G in a humidified incubator (Heraeus Hera Cell incubator) at 37 °C with 5% CO_2_. They were seeded in 96-well microtiter plates at 1 × 10^4^ cells/well and allowed to adhere for 24 h in the incubator. The medium was removed, and adherent cells were washed with PBS. Fresh media containing serially diluted peptides in PBS (final concentrations 4 to 250 µg/mL) were added and the cells incubated for 24 h. Cells without peptides were used as negative controls. Following incubations, the medium was removed, and adherent cells were washed with PBS. 150 µL of MTT (diluted to 0.33 mg/mL in DMEM) and the plates incubated for 3 h. The precipitated formazan was dissolved in 100 µL of DMSO and its absorbance measured at 570 nm. Cell viability was determined as: (OD_570 nm_ peptide treated cells/(OD_570 nm_ untreated cells) × 100. Positive cytotoxicity was defined as peptide concentration that causes 50% growth inhibition in a cell line (IC_50_). All tests were carried out in triplicate.

### 4.16. Scanning Electron Microscopy

SEM of cells was carried out as described by Tripathi et al. [74], with some modifications. Briefly, cultures of MRSA M173525 were grown overnight at 37 °C in MHB, then adjusted to 1 × 10^8^ CFU/mL (0.5 McFarland). A total of 75 μL aliquots of the cell suspension were incubated with 25 μL of P1 or PuroA (final concentrations of 16 μg/mL, based on MIC) for 3 h at 37 °C. The cells were collected by centrifugation at 1000× *g* for 2 min, washed twice with 0.1 M PBS and resuspended in 50 μL PBS. 20 μL aliquots of cell suspensions were spotted onto clean glass slides and air-dried. The slides were fixed in 2.5% glutaraldehyde (Sigma) in PBS for 2h in a humid chamber (a Petri dish containing a filter paper soaked in sterile water), then washed with 0.1 M PBS for 10 min and progressively dehydrated in ethanol (50%, 60%, 70%, 80%, 90%, 100%). The slides were freeze-dried overnight and coated in Dynavac CS300 with gold, followed by attachment of double-sided conducting carbon tape to the slide for better conductivity. The samples were analysed using a ZEISS supra 10 VP field emission SEM (Carl Zeiss, NY, USA) and images captured. HeLa cells were seeded at 2 × 10^4^ cells on a coverslip and allowed to adhere for 24 h at 37 °C with a 5% CO_2,_ then washed with PBS. Fresh media containing peptides P1 and PuroA (final concentration 64 and 125 µg/mL) were added and the cells incubated for further 24 h. Cells treated with media without peptides were used as negative controls. The cells were washed with PBS, then fixed and observed by SEM as above.

### 4.17. Statistical Analysis

All experiments were performed at least in triplicate and data are presented as mean values. The inhibitory activity of peptides on biofilm formation was found to be non-normally distributed using the Kolmogorov and Smirnov test; hence, the non-parametric Kruskal–Wallis test and a Dunn’s post-hoc test were used to identify the differences between peptide-treated and untreated samples. SPSS Statistics v23 (IBM) was used for analysis and differences with *p* < 0.05 were considered as statistically significant.

## 5. Conclusions

The PuroA based peptides P1, W7, W8, and WW displayed potent antimicrobial activities and stability under physiological salt conditions. The diastereomers, dP1, dW7 and dWW were antimicrobial and resistant to in vitro proteolytic degradation. Further, P1 and Di-PuroA showed high selectivity towards HeLa cells, suggesting their potential as anticancer peptides. The data confirm that consideration of the diverse and interacting physico-chemical factors can assist significantly in designing peptides with unique bioactivities. Further optimisation of peptide structures and investigations of the mechanism of action would pave the way for designing peptides as novel antibacterial, anti-viral or anticancer agents, to address global challenges in healthcare, hygiene and/or food safety.

## Figures and Tables

**Table 1:** Physico-chemical properties of the designed Trp-rich peptides (TRPs).

Peptide ID	Sequences *	Length	Mw	Number of Trp	Net Charge	Isoelectic Point (pI)	Charge Density (ChD)	Hydrophobicity (H)	Hydropobic Ratio (HR)	GRAVY	Hydrophobic Moment (µH)
**PuroA**	FPVTWRWWKWWKG-NH_2_	13	1863	5	+3	11.17	0.23	−0.08	61.5	−0.962	5.21
**Cyclic PuroA**	FPVTWRWWKWWKG	13	1845	5	+3	11.17	0.23	−0.08	61.5	−0.962	5.21
**Di-PuroA**	(FPVTWRWWKWWKG)_2_k-NH_2_	27	3836	10	+7	12.04	0.25	−0.11	59	−1.070	3.56
**PuroA-OH**	FPVTWRWWKWWKG	13	1863	5	+3	11.17	0.23	−0.08	61.5	−0.962	5.21
**R8**	RRRRWRWWRWWRR-NH_2_	13	2198	5	+8	12.85	0.61	−0.94	38	−3.115	5.01
**P1**	RKRWWRWWKWWKR-NH_2_	14	2144	6	+7	12.48	0.5	−0.58	43	−2.700	7.88
**dP1**	RK**R**WW**R**W**WK**WW**K**R-NH_2_	14	2144	6	+7	12.48	0.5	ND	43	ND	ND
**R6**	RWWKWW-NH_2_	6	1047	4	+2	11.00	0.33	−0.23	66	−2.000	5.56
**R7**	RWWKWWK-NH_2_	7	1175	4	+3	11.17	0.42	−0.35	57	−2.271	6.01
**W7**	WRWWKWW-NH_2_	7	1233	5	+2	11.00	0.28	−0.14	71	−1.843	4.51
**dW7**	W**R**W**WK**WW-NH_2_	7	1233	5	+2	11.00	0.28	ND	71	ND	ND
**W8**	WRWWKWWK-NH_2_	8	1361	5	+3	11.17	0.37	−0.26	62.5	−2.100	4.71
**WW**	WWRWWKWW-NH_2_	8	1419	6	+2	11.00	0.25	−0.08	75	−1.725	6.32
**dWW**	WW**R**W**WK**WW-NH_2_	8	1419	6	+2	11.00	0.25	ND	75	ND	ND

* Underlined and bold amino acids are D-enantiomers. The C terminus is amidated except for PuroA-OH peptide. ND: not determined. GRAVY: grand average of hydropathy.

**Table 2:** Antimicrobial activities of peptides.

Peptide ID	Sequence	MIC (μg/mL) *					
		*E. coli* ATCC25922	*S. aureus* ATCC25923	*P. aeruginosa* ATCC9027	*C. albicans* FRR 5580	MRSA ** M173525	MRSA ** M180920
PuroA	FPVTWRWWKWWKG-NH_2_	16	16	64	125	16	16
Cyclic PuroA	FPVTWRWWKWWKG	250	125	>250	>250	N/T	N/T
Di-PuroA	(FPVTWRWWKWWKG)_2_k-NH_2_	250	250	>250	125	N/T	N/T
PuroA-OH	FPVTWRWWKWWKG	32	64	125	250	N/T	N/T
R8	RRRRWRWWRWWRR-NH_2_	64	64	64	250	N/T	N/T
P1	RKRWWRWWKWWKR-NH_2_	16	16	16	64	16	8
dP1	RKRWWRWWKWWKR-NH_2_	16	16	16	64	N/T	N/T
R6	RWWKWW-NH_2_	32	16	32	64	N/T	N/T
R7	RWWKWWK-NH_2_	8	16	32	64	N/T	N/T
W7	WRWWKWW-NH_2_	4	8	32	64	8	8
dW7	WRWWKWW-NH_2_	8	8	16	64	N/T	N/T
W8	WRWWKWWK-NH_2_	4	8	32	64	N/T	N/T
WW	WWRWWKWW-NH_2_	8	8	32	64	4	4
dWW	WWRWWKWW-NH_2_	8	8	32	64	N/T	N/T

Minimum inhibitory concentration (MIC) was determined as the lowest concentration of peptide that inhibited bacterial or yeast growth. * Data are mode of three independent experiments; ** Methicillin resistant *Staphylococcus aureus*; N/T: not tested.

**Table 3:** Salt tolerance, protease stability and DNA binding properties of peptides.

Peptide ID	Salt Tolerance *	Protease Stability **	Binding to DNA in vitro (µg/mL)
PuroA	Up to 50mM	Unstable	≥32
Cyclic PuroA	Intolerant	Unstable	≥64
Di-PuroA	Intolerant	Unstable	≥8
PuroA-OH	Intolerant	Unstable	≥32
R8	Up to 150 mM	Unstable	≥64
P1	Up to 150 mM	Unstable	≥8
dP1	Up to 150 mM	Stable	≥8
R6	Intolerant	Unstable	≥250
R7	Intolerant	Unstable	≥250
W7	Up to 150 mM	Unstable	≥16
dw7	Up to 150 mM	Stable	≥32
W8	Up to 150 mM	Unstable	≥16
WW	Up to 150mM	Unstable	≥32
dWW	Up to 150 mM	Stable	≥32

* Up to 50 mM: no effects on antibacterial activity up to 50 mM NaCl but increase in the MICs for *E. coli* and *S. aureus* at 100 mM NaCl; intolerant = not resistant to salt effects even at 50 mM NaCl. Up to 150 mM: no effects on antibacterial activity up to 150 mM NaCl. ** Proteases tested: porcine trypsin and proteinase K.

**Table 4:** Effects of peptides on initial cell attachment and preformed biofilms of MRSA M173525.

Peptide ID	Peptide Concentration	Percentage of Inhibition of Initial MRSA Cells Attachment		% Inhibition of Preformed 6 h MRSA Biofilm after 1 h Incubation	
		CV	MTT	CV	MTT
**PuroA**	0.5 × MIC	70 ± 5.6	92 ± 1.5	68 ± 9.2	90 ± 2.5
	1 × MIC	75 ± 4.0	95 ± 1.1	75 ± 7.5	92 ± 4.0
	2 × MIC	79 ± 4.5	95 ± 0.5	77 ± 7.0	95 ± 2.6
**P1**	0.5 × MIC	72 ± 3.1	92 ± 3.0	70 ± 6.9	93 ± 5.0
	1 × MIC	75 ± 1.9	95 ± 2.3	74 ± 5.8	95 ± 3.8
	2 × MIC	78 ± 6.1	95 ± 2.5	78 ± 3.1	95 ± 4.0
**W7**	0.5 × MIC	69 ± 3.6	90 ± 2.9	67 ± 6.0	88 ± 2.5
	1 × MIC	75 ± 5.0	95 ± 4.0	75.5 ± 3.0	90 ± 4.7
	2 × MIC	76 ± 4.8	96 ± 1.5	75 ± 5.5	94 ± 0.5
**WW**	0.5 × MIC	71.5 ± 3.5	90 ± 2.0	69 ± 7.8	91 ± 3.0
	1 × MIC	73 ± 6.2	95 ± 3.1	71 ± 4.5	92 ± 3.5
	2 × MIC	79 ± 7.5	96 ± 0.5	74 ± 2.0	95 ± 1.7

**Table 5:** Cytotoxicity of peptides to NIH-3T3 and HeLa cells.

Peptide ID	* IC_50_ (μg/mL)		** Therapeutic Index (TI)
	NIH-3T3	HeLa	
PuroA	250	>250	<1
Cyclic PuroA	>250	>250	ND
Di-PuroA	250	32	7.8
PuroA-OH	>250	>250	ND
R8	>250	250	>1
P1	250	32	7.8
dP1	250	125	2
R6	250	>250	<1
R7	250	>250	<1
W7	250	250	2
dW7	>250	125	>3
W8	>250	250	>2
WW	>250	250	>2
dWW	250	125	2

* IC_50_: concentration causing 50% cell growth inhibition. ** Therapeutic index = ratio of IC_50_ NIH-T3T (μg/mL) to IC_50_ HeLa (μg/mL) [34]. ND = not determined. “<” indicates the maximum TI calculated with 250 μg/mL. “>” indicates the minimum TI calculated with 250 μg mL^−1^, i.e., no IC_50_ values can be calculated within the tested concentration range of peptides (0–250 μg/mL). Results are the mean of triplicate assays.

## Supplementary Materials

Supplementary Materials can be found at https://www.mdpi.com/1422-0067/21/22/8624/s1.

## Abbreviations

AMP	antimicrobial peptide(s)
CD	circular dichroism
SEM	scanning electron microscopy
pI	isoelectric point
ChD	charge density
H	hydrophobicity
HR	hydrophobic ratio
µH	hydrophobic moment
MIC	Minimum Inhibitory Concentration
CV	crystal violet
MTT	methylthiazolyldiphenyl-tetrazolium
