# Supplementary material for: Effects of Rationally Designed Physico-Chemical Variants of the Peptide PuroA on Biocidal Activity towards Bacterial and Mammalian Cells
Source: Int J Mol Sci. 2020 Nov 16;21(22):8624. doi: 10.3390/ijms21228624 (PMC7696940; doi:10.3390/ijms21228624)
Supplement: Supplementary file 1 [file ijms-21-08624-s001.pdf]

SUPPLEMENTARY DATA

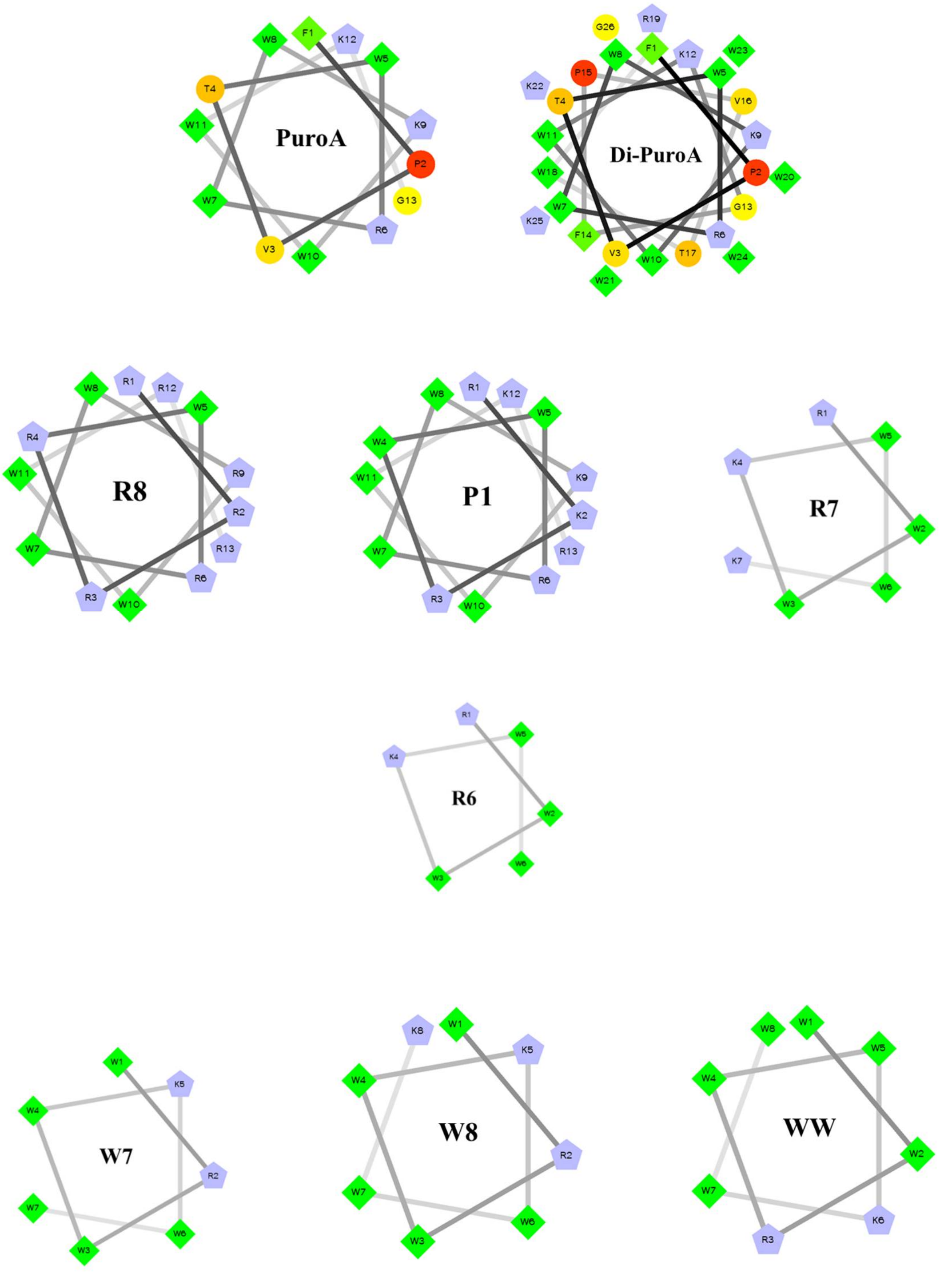

Figure S1. Helical wheel projections of peptides.

Residues in circles are hydrophilic, diamonds are hydrophobic, and pentagons are potentially positively charged residues. Green: most hydrophobic residues; yellow: residues with zero hydrophobicity; red: most hydrophilic (uncharged) residues; light blue: potentially charged residues

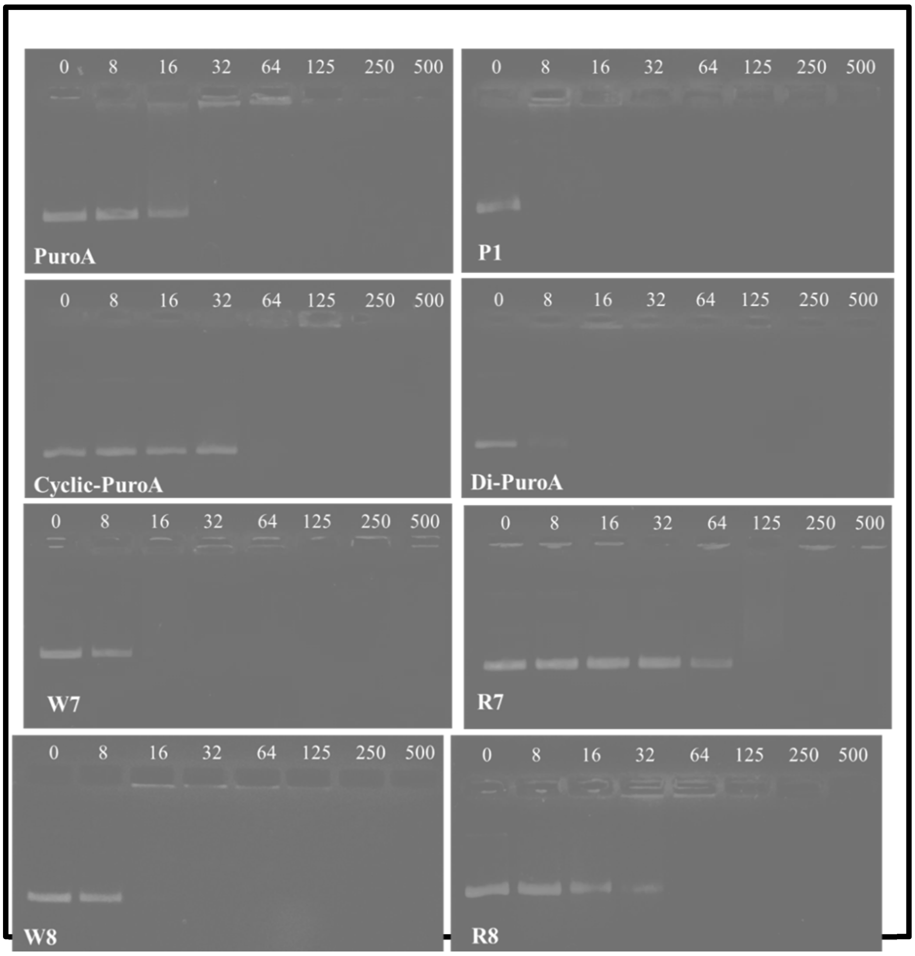

Figure S2. Plasmid DNA gel retardation by peptides. 100 ng of purified pBluescript SK(+) plasmid DNA was mixed with peptides to final concentrations of 0, 8, 16, 32, 64, 125, 250 and 500 µg/mL before electrophoresis.

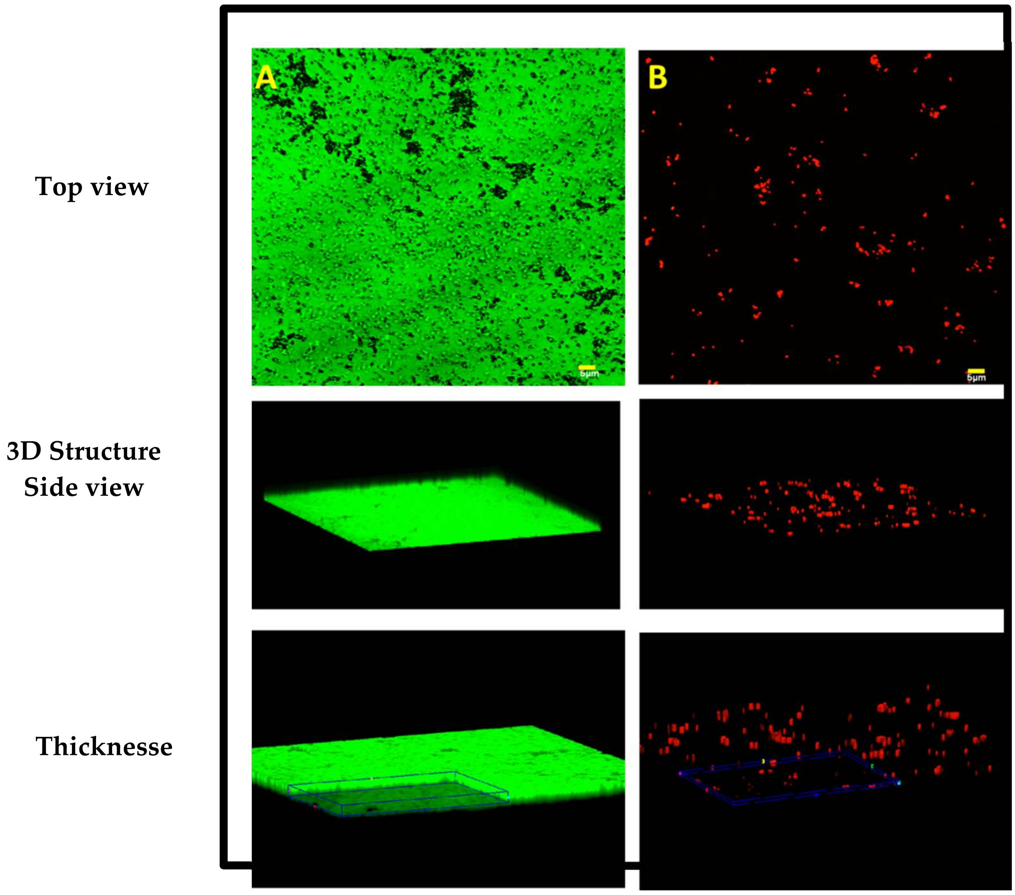

Figure S3. Effects of P1 on initial adhesion and biofilm formation of the clinical MRSA M173525 isolate. The biofilms were visualized by confocal laser scanning microscopy with the Live/Dead viability stain (SYTO9/PI); viable cells exhibit green fluorescence, whereas dead cells exhibit red fluorescence. **A:** no-peptide control; **B:** P1 (final concentration 16 μg/mL). Magnification 1,000×, scale bar 5 μm

Table S1

**Peptide ID**	**MIC (μg mL^{-1})***							
	**No NaCl**		**With 50mM NaCl**		**With 100mM NaCl**		**With 150mM NaCl**	
	E. coli	*S. aureus*	*E. coli*	*S. aureus*	*E. coli*	*S. aureus*	*E. coli*	*S. aureus*
**PuroA**	16	16	16	16	32	32	64	64
**Cyclic PuroA**	250	125	> 250	250	>250	250	>250	>250
**Di-PuroA**	250	250	> 250	> 250	>250	>250	>250	>250
**PuroA-OH**	32	64	64	125	>250	>250	>250	>250
**R8**	64	64	64	64	64	64	64	64
**P1**	16	16	16	16	16	16	16	16
**dP1**	16	16	16	16	16	16	16	16

**R6**	32	16	64	32	250	250	>250	>250
**R7**	8	16	32	32	250	250	>250	>250
**W7**	4	8	4	8	4	8	4	8
**dW7**	8	8	8	8	8	8	8	8
**W8**	4	8	4	8	4	8	4	8
**WW**	8	8	8	8	8	8	8	8
**dWW**	8	8	8	8	8	8	8	8
